# Tubular Pregnancy

**Published:** 1870-06

**Authors:** W. C. Hunt

**Affiliations:** Chicago


### Article II.—*Tubular Pregnancy.* By W. C. Hunt, M. D., Chicago.

Nov. 12th, 1869, I was called to see Mrs. H.

Her menses had been regular from the birth of her last child, nine years before, until about a month previous to my being called. They had then ceased, some of the usual symptoms of pregnancy immediately following. The main object of my being called was, to relieve, if possible, the nausea and a very distressing sense of impending syncope, which threatened upon the slightest exertion.

Upon the supposition that she was pregnant, little was done, except to relieve (temporarily) the nausea and faintness. Two weeks after this I was again called. The menses were apparently re-established, but very scanty. The tendency to syncope had not abated, and the patient's anxiety was, the fear of "heart disease." "Two of her near relatives had suddenly died of it." During the following week, another slight hemorrhage from the uterus occurred, which cast doubt upon the previous diagnosis, and from this time forward, during the whole of her illness, the hemorrhage continued at intervals of from three days to a week.

At this time, Prof. Freer (who was the family physician) was called in, and an examination revealed the existence of a tumor of the size of a man's fist in the right upper portion of the hypogastric region. But little attention was paid to this, for from this time, an entirely new and different set of symptoms supervened, which masked the whole character of the case. At intervals of three or four days, paroxysms of pain in the right hypochondriac and epigastric region would come on suddenly and violently, continuing for two or three hours, and ending in complete syncope. With recovery from the syncope, came entire relief of all the symptoms, except a yellowness of the skin, which followed these attacks, to disappear usually at the time of the next.

The passage of stones from the gall bladder was diagnosed.

The tumor was suspected to be in the ovary. In this condition the patient continued, alternating between the pain and syncope, and gradually improving during the interval, without much loss of strength when the pain was absent, until the 14th of January, 1870, when, after an unusually severe attack of the pain, she died in the syncope that followed.

Autopsy: 30 hours after death. Present, Profs. Freer and Blaney, and Dr. Parkes.

Skin, mucous membrane and conjunctiva intensely jaundiced.

Cavity of the abdomen filled with blood, of which 2½ gallons were dipped out.

Liver pale, exsanguine; gall bladder entirely empty, large and flabby.

The right Fallopian tube contained a fœtus of three months growth. The placenta attached at or near the junction of the tube with the uterus—a portion of its attachment had separated, and from this the fatal hemorrhage had occurred, rupturing the distended walls of the tube, and escaping into the abdominal cavity. This attachment of the placenta was the source of the frequent hemorrhages from the uterus during the last two months.

Note. The uterus and appendages taken from the above case may be seen in the museum of the Rush Medical College.

